# Modelling the mechanoreceptor's dynamic behaviour

**DOI:** 10.1186/1471-2202-16-S1-P16

**Published:** 2015-12-18

**Authors:** Zhuoyi Song, Robert W Banks, Guy S Bewick

**Affiliations:** 1 Centre for Mathematic, Physics and Engineering in the Life Sciences and Experimental Biology (CoMPLEX), University College London, London, UK; 2 School of Biological and Biomedical Sciences, University of Durham, Durham, DH1 3LE, UK; 3 School of Medical Sciences, Institute of Medical Sciences, University of Aberdeen, Aberdeen, AB25 2ZD, UK

## 

All sensory receptors adapt, i.e., they constantly adjust their sensitivity to external stimuli to match the current natural environment [1]. Electrophysiological responses of sensory receptors from widely different modalities seem to exhibit common features related to adaptation, and these features can be used to examine the underlying sensory transduction mechanisms [1,2]. Among the principal senses, mechanosensation remains the least understood at the cellular level [3]. To gain greater insights into mechanosensory signalling, we investigated if mechanosensation displayed adaptive dynamics that could be explained by similar biophysical mechanisms in other sensory modalities. To do this, we adapted a fly photoreceptor model [4] to describe the primary transduction process for a stretch-sensitive mechanoreceptor, taking into account the viscoelastic properties of the accessory muscle fibres [5] and the biophysical properties of known mechanosensitive channels (MSCs). The model's output is in remarkable agreement with the electrical properties of a primary ending of an isolated decapsulated spindle; ramp-and-hold stretch evokes a characteristic pattern of potential change, consisting of a large dynamic depolarization during the ramp phase and a smaller static depolarization during the hold phase [6]. The initial dynamic component is likely to be caused by both the mechanical properties of the muscle fibres and a refractory state of MSCs. Consistent with literature, the current model predicts that the dynamical component is due to a rapid stress increase during the ramp [7]. More novel predictions from the model are the mechanisms to explain the initial peak in the dynamical component. At the onset of the ramp, all MSCs are sensitive to external stimuli, but as they become refractory (clipped inactivated state), fewer MSCs are able to respond to the continuous stretch, causing a sharp decrease after the peak response. The same mechanism could contribute a faster component in 'sensory habituation' of a mechanoreceptor, in which a receptor responds more strongly to the first stimulus episode during repetitive stimulation [8].
